# Left-handedness and risk of breast cancer

**DOI:** 10.1038/sj.bjc.6603920

**Published:** 2007-08-07

**Authors:** L Fritschi, M Divitini, A Talbot-Smith, M Knuiman

**Affiliations:** 1 Western Australian Institute for Medical Research, Nedlands, Perth, Western Australia 6009, Australia; 2 School of Population Health, University of Western Australia, Nedlands, Perth, Western Australia, Australia; 3 Hereford County Hospital, Hereford, UK

**Keywords:** breast cancer, left-handedness, fetal origins of disease, cohort

## Abstract

Left-handedness may be an indicator of intrauterine exposure to oestrogens, which may increase the risk of breast cancer. Women (*n*=1786) from a 1981 health survey in Busselton were followed up using death and cancer registries. Left-handers had higher risk of breast cancer than right-handers and the effect was greater for post-menopausal breast cancer (hazard ratio=2.59, 95% confidence interval 1.11–6.03).

Intrauterine exposure to oestrogens has been hypothesised as being a risk factor for breast cancer (Trichopoulos, 1990). Left-handedness has been linked to high fetal exposure to oestrogens on the basis of left-handedness being associated with diethylstilbestrol exposure (Schachter, 1994; Scheirs and Vingerhoets, 1995). Left-handedness may therefore be thought of as a proxy for high intrauterine exposure to oestrogens. In a recent paper, a modest association was reported between left-handedness and breast cancer risk (adjusted hazard ratio (HR)=1.32, 95% confidence interval (CI) 0.99–1.76) in a case-cohort study; (Ramadhani *et al*, 2005). They found that the association was confined to pre-menopausal breast cancer. An earlier case–control study (Titus-Ernstoff *et al*, 2000) had found a similar effect but in post-menopausal women (OR=1.42, 95% CI 1.10–1.83). We examined this question in a prospective study in Australia.

## MATERIALS AND METHODS

Between 1966 and 1981, the Australian town of Busselton was the site of triennial cross-sectional health surveys consisting of a general health questionnaire (including a question on handedness) and various health-related tests. In the 1981 survey, 3940 adults participated (64% of those eligible), with 14 subsequently excluded owing to a diagnosis of malignancy before the survey date. The cohort was followed to the end of 2004. Linkage to death registrations was used to identify deaths occurring within Western Australia. Information from relatives and linkage to electoral rolls was used to identify if and when participants had left Western Australia. With cancer notification mandatory in Australia, linkage to the Western Australian Cancer Registry was used to determine the incidence of specific cancers.

Relative risk estimates (HRs) for left-handedness in relation to development of breast cancer were calculated from Cox proportional hazards regression models that also adjusted for age, smoking category, body mass index, number of pregnancies and menopausal status at the 1981 survey. We also fitted two further models that limited person-time at risk to pre-menopausal years (up to age 51 years for women who were pre-menopausal in 1981) and to post-menopausal years (over age 51 years).

## RESULTS

Among the 1786 women who participated in the survey and were not excluded owing to having missing data, there were a total of 94 cases of primary breast cancer diagnosed during the follow-up period. Of 1637 right-handers, 86 (5.3%) developed breast cancer compared with 7 (7.5%) of 93 left-handers and 1 (1.8%) of 56 women who had reported being ambidextrous. Twenty-two breast cancer cases were diagnosed before the age of 51 (average age of menopause in Australia) and 72 cases were diagnosed after the age of 51 years.

After adjusting for the other variables, left-handers were more likely to develop breast cancer than right-handers, but the confidence interval was wide (HR=1.71, 95% CI 0.79–3.74). The risk of developing pre-menopausal breast cancer was lower for left-handed women compared with those who were right-handed or ambidextrous, but this was not statistically significant (HR=0.64, 95% CI 0.09–4.82). The risk of developing post-menopausal breast cancer was significantly higher for left-handers compared to right-handers, (HR=2.59, 95% CI 1.11–6.03).

## DISCUSSION

These results are based on very small numbers and the CIs are wide. However, they are consistent with the previous finding from a case–control study of an increase in the risk of breast cancer which is confined to post-menopausal breast cancer (Titus-Ernstoff *et al*, 2000). Because of the implications for the theory of causation of breast cancer, more studies are needed, and future studies should examine pre- and post-menopausal breast cancer separately.
